# Nucleotide-binding oligomerization domain 1 (NOD1) regulates microglial activation in pseudorabies virus infection

**DOI:** 10.1186/s13567-024-01416-5

**Published:** 2024-12-18

**Authors:** Xiuxiu Sun, Xinxin Jin, Zhengdan Lin, Xi Liu, Junjie Yang, Li Li, Helong Feng, Wanpo Zhang, Changqin Gu, Xueying Hu, Xiaoli Liu, Guofu Cheng

**Affiliations:** 1 Division of Veterinary Pathology, College of Veterinary Medicine, Huazhong Agricultural University, Wuhan, China; 2 Institute of Animal Health and Veterinary Sciences, Hubei Academy of Agricultural Sciences, Wuhan, China

**Keywords:** Microglia, neuroinflammation, pseudorabies virus, NOD1, JNK, NF-κB

## Abstract

The primary cause of viral encephalitis (VE) is invasion of the central nervous system (CNS) by the virus, which leads to neuroinflammation and poses a significant threat to global public health. Microglia, as CNS-resident macrophages, play a crucial role in neuroinflammation and are often identified as the preferred target for the prevention or treatment of VE. In this study, we used pseudorabies virus (PRV)-induced VE in mice and pigs as a model to investigate the regulation of microglial responses during viral encephalitis and explored the mechanism of microglial activation. Cellular experiments revealed that microglial activation was accompanied by cell migration, characteristic morphological changes, phagocytosis, inflammatory cytokine production, and antigen presentation. Transcriptome analysis revealed that genes related to inflammation in PRV-infected BV2 cells were significantly enriched. The expression of the NOD1 gene in BV2 cells was significantly increased during PRV infection, after which NOD1 in BV2 cells was silenced by siRNA and overexpressed via a plasmid. NOD1 was found to be involved in the secretion of cytokines in BV2 cells by regulating the MAPK/NF-κB signalling pathway. Mouse and pig experiments have shown that NOD1 is involved in the secretion of cytokines by microglia by regulating the MAPK/NF-κB signalling pathway during PRV infection.

## Introduction

Pseudorabies virus (PRV) is an enveloped 140 kb, double-stranded linear DNA virus belonging to the *Herpesviridae* family, *α-Herpesvirinae* subfamily, and *Varicellovirus* genus [1, 2]. Like herpes simplex virus type 1 (HSV-1) and rabies virus (RABV) [3, 4], PRV envelope glycoproteins fuse with the host cell membrane, allowing the virus to invade the central nervous system (CNS), to replicate and release viral particles, causing fatal neuroinflammation characterised by neuronal destruction, microgliosis, astrogliosis, and the production of various inflammatory cytokines [5, 6]. Recent reports indicate direct PRV infection in humans, resulting in severe damage to the nervous and respiratory systems [7–10]. Innate immunity within the CNS is a crucial first line of defence against virus infection and involves microglial activation and peripheral immune cell infiltration [11].

Microglia, as resident myeloid cells in the CNS, mediate innate immune responses and can transition from a resting to an activated form upon sensing environmental danger signals [12, 13]. However, hyperactivated microglia can release inflammatory cytokines such as IL-1β, IL-6, iNOS, COX-2, and TNF-α, contributing to brain inflammation [14–16]. Therefore, inhibiting the overactivation of microglia has become an important direction in the treatment of viral encephalitis.

Nucleotide-binding oligomerization domain 1 (NOD1) belongs to the NOD-like receptor (NLR) family of pattern recognition receptors (PRRs), which recognize pathogen-associated molecular pattern (PAMP) molecules and damage-associated molecular pattern (DAMP) molecules, thus activating innate immune responses such as the engulfment of invading viruses by macrophages, the suppression of viral replication by interferon-gamma, and the destruction of infected cells by natural killer cells [17–19]. Previous research has indicated that NOD1 positively regulates neuroinflammation caused by JEV infection and that inhibiting NOD1 exerts a neuroprotective effect [20]. NOD1 can be activated by hepatitis C virus (HCV) polymerase to regulate innate immune responses and inflammation [21, 22]. Receptor-interacting serine/threonine kinase 2 (RIP2/RIPK2) is the receptor-interacting protein of NOD1 [23]. The NOD1-RIPK2 interaction induces the activation of nuclear factor-kappa B (NF-κB) and mitogen-activated protein kinase (MAPK) signalling cascades, contributing to inflammatory gene activation [24, 25]. These findings emphasize the critical role of NOD1/RIPK2 signalling in the inflammatory response. We aimed to investigate whether NOD1/RIPK2 signalling is involved in the pathogenesis of PRV infection-induced neuroinflammation, particularly its mediation of microglial activation and subsequent inflammatory signalling during PRV infection.

This study revealed that the expression of NOD1 and RIPK2 is upregulated in BV2 cells and mouse brain tissues during PRV infection, which may be influenced by other signalling pathways after infection, and further research is needed. Furthermore, NOD1 overexpression or inhibition regulates microglial activation and cytokine production by modulating the NF-κB and MAPK signalling pathways both in vitro and in vivo. Consequently, inhibiting microglial activation and subsequent neuroinflammation by regulating NOD1/RIPK2 may be a potential therapeutic strategy for PRV-induced neuronal injury.

## Materials and methods

### Ethics approval and consent to participate

All the mice used in this study were treated humanely during the experiment and euthanized at the end of the experiment. The animal care and use protocols used were reviewed and approved by the experimental animal monitoring committee of Huazhong Agricultural University (approval number: HZAUMO-2023–0142).

### Cells and virus

Mouse microglia (BV2) and Neuro-2a (N2a) cells were cultured in Dulbecco’s modified Eagle’s medium (DMEM) supplemented with 10% fetal bovine serum (FBS), 1% l-glutamine, 100 U/mL penicillin, and 100 mg/mL streptomycin at 37 °C with 5% CO_2_.

PRV was generously provided by Professor Bin Wu (Huazhong Agricultural University). The viruses were propagated in a porcine kidney cell line (PK-15) and cultured in DMEM supplemented with 2% FBS at 37 °C with 5% CO_2_ in a humidified incubator.

The Transwell migration experiments followed basic procedures: N2a cells were placed in the lower chamber and exposed to PRV (10^4^ TCID_50_) or the control. BV2 cells were subsequently placed in the upper transwell chamber. After 24 h of incubation, the migrated BV2 cells on the lower surface of the filter membrane were fixed with 4% paraformaldehyde for 20 min and stained with 0.1% crystal violet for 20 min at room temperature. The number of migrating BV2 cells was determined under a microscope.

### Animal experiments

Six-week-old female BALB/c mice were procured from the Laboratory Animal Center of Huazhong Agricultural University (Wuhan, China). All mouse procedures received approval from the Institutional Animal Care and Use Committee of Huazhong Agricultural University (approval number: HZAUMO-2023–0142). The mice were housed in a strictly pathogen-free environment under a 12-h light/dark cycle with free access to water. The mice were randomly allocated into four groups: mock, ML130, PRV-infected mock (PRV), and PRV-infected ML130 (PRV + ML130) groups. Daily monitoring of behavior and mortality was conducted for all groups. Individual mice were subsequently euthanized, and brain tissue samples were collected for further experiments.

Twelve 90-day-old PRV antigen- and antibody-negative pigs were randomly divided into 4 groups (GP1‒GP4). Each pig in the GP4 group was intranasally infected with 2 mL of DMEM, and each pig in the GP1‒GP3 group was intranasally infected with 2 × 10^7^ TCID_50_ PRV. The incidence of the disease was observed every day. When the pigs began to die, all the pigs in the same group were dissected, and the brain tissues were collected. All pig procedures received approval from the Institutional Animal Care and Use Committee of Huazhong Agricultural University (approval number: HZAUSW-2022-0018) [26].

### Antibodies and reagents

Anti-GFAP (80788), anti-MHCI (79769), anti-MHCII (86285), anti-NOD1 (3545), anti-RIPK2 (4142), anti-IκBα (4812), anti-NF-κB (8242), anti-p-NF-κB (3033), anti-JNK (9252), anti-p-JNK (4668), anti-P38 (9212), and anti-p-P38 (4511) antibodies were obtained from Cell Signaling Technology. Anti-β-actin (AC026) and anti-NOD2 (A15992) antibodies were purchased from ABclonal Technology Co., Ltd., Wuhan, China. ML130, a highly selective and effective NOD1 inhibitor (20 µM in vivo and in vitro), was acquired from Selleck Chemicals.

### Flow cytometry analysis

Mouse microglia were obtained via Percoll gradient centrifugation and incubated with APC-anti-CD11b, FITC-anti-CD45, PC5.5-anti-MHCI and PE-anti-MHCII at 4 °C for 30 min. Then, 1 mL of PBS containing 2% BSA was added to resuspend and mix well, the mixture was centrifuged at 1000 r/min for 5 min, and the mixture was washed 30 times. Then, 500 μL of 0.2% BSA in PBS was added, the mixture was subjected to flow cytometry, and FlowJo was used to analyse the data.

### Cell proliferation assay

The cells were resuspended in medium containing 2% FBS, and the cell density was adjusted to N2a (5 × 10^4^ cells/well) and BV2 (2 × 10^4^ cells/well). One milliliter of N2a cell suspension was added to the lower chamber of the Transwell system, and after 1 day of adherent growth, 0.1 MOI PRV was inoculated. Two hundred microliters of the BV2 cell suspension was added to the upper chamber of a Transwell and placed in an empty 12-well plate. After 1 day of adherent growth, the cell plate was placed in the incubator for 48 h. The chamber was fixed with 4% paraformaldehyde fixative for 25 min. The fixed chamber was removed and washed with PBS once. The nonmigrating cells in the upper layer of the chamber membrane were gently removed with a cotton swab, and the migrating cells in the lower layer of the chamber membrane were stained with Gram’s staining solution for 2 min.

### Plasmid construction

NOD1 was cloned by PCR from total cDNA (GenBank accession No. NM_001171007.1) and inserted into pcDNA3.1 (Invitrogen). The recombinant plasmid was verified by sequencing. NOD1 overexpression was confirmed in transduced BV2 cells by assessing the mRNA and protein levels.

### RNA interference

Mouse NOD1-specific siRNAs were designed via the RNAi Target Sequence Selector website (Clontech). The sequences of the siRNAs used for NOD1 are shown in Table 1. BV2 cells were plated in 24-well plates, cultured overnight, and transfected with siRNAs for 24 h. Knockdown efficiency was assessed via qPCR and WB after 24 h. BV2 cells were transfected with negative-control siRNA (siNC) or the indicated siRNAs for 24 h and then infected with PRV (MOI = 0.1). After another 24 h of infection, the cell lysates were harvested and assessed.

**Table 1:** **siRNA sequences of the NOD1 genes**

Gene	siRNA sequence
NOD1-siRNA1 sense	CAGGGCCAGUCUUACGAAUUUTT
NOD1-siRNA1 antisense	AAAUUCGUAAGACUGGCCCUGTT
NOD2-siRNA1 sense	CAAGUAUAAGAUCGUGACGUUTT
NOD2-siRNA1 antisense	CACGAUCUUAUACUUGTT
NOD3-siRNA1 sense	GCUCUUCUGUUGGAUCAUCUUTT
NOD3-siRNA1 antisense	AAGAUGAUCCAACAGAAGAGCTT
NOD4-siRNA1 sense	ACUCCCACAUUAAACUGCUGATT
NOD4-siRNA1 antisense	AUCUUCAGCAGUUUAAUGUGGTT
NOD5-siRNA1 sense	CAGGGAACAUCUGGUCACCAATT
NOD5-siRNA1 antisense	AAUGUUGGUGACCAGAUGUUCTT

### Enzyme-linked immunosorbent assays (ELISA)

Inflammatory cytokine levels in the cell supernatant were measured using ELISA. Specific steps were conducted following the manufacturer’s instructions. Inflammatory cytokine release was measured in supernatants from the same cells used for RNA or protein isolation.

### Western blot (WB) analyses

Brain tissues or cultured cells were lysed with cold RIPA lysis buffer and centrifuged. The supernatant was collected. The protein concentration was determined via a BCA protein assay kit. The samples were boiled with 5 × loading buffer, separated via SDS–PAGE, transferred onto a polyvinylidene difluoride (PVDF) membrane, blocked with 5% skim milk for 2 h, and then incubated with the corresponding primary antibody overnight at 4 °C. The next day, the membrane was incubated with the secondary antibody and visualized via an enhanced chemiluminescence (ECL) detection reagent in a chemiluminescence imaging system. Images were saved for analysis.

### Haematoxylin and eosin (HE) staining

The brain tissues were fixed in 10% formalin-neutral buffer and embedded in paraffin. Then, the tissues were dehydrated via a series of procedures, embedded in paraffin blocks, cut into 3-µm sections, and stained with H&E. The results were imaged at × 20 magnification via a bright-field microscope.

### Immunofluorescence (IF) analysis

Paraffin-embedded brain tissue sections were deparaffinized with xylene and a gradient of alcohol and water. Heat retrieval was carried out via sodium citrate buffer (pH 6) for 15 min. The sections were then blocked with goat serum at room temperature for 30 min, incubated with the primary antibody overnight at 4 °C, and exposed to a fluorescent secondary antibody at room temperature for 1 h. Subsequently, the sections were stained with 4′,6-diamidino-2-phenylindole (DAPI) for 10 min. Cultured cells were fixed with 4% paraformaldehyde for 30 min, permeabilized with 1% Triton X-100 for 10 min, blocked with goat serum for 30 min, incubated with the primary antibody overnight at 4 °C, and then incubated with a fluorescent secondary antibody at room temperature for 1 h. Finally, the cells were stained with DAPI for 10 min. The samples were observed and photographed under a fluorescence microscope.

### Statistical analysis

All the data are presented as the means ± SD. Statistical analysis was performed using GraphPad Prism 8.0 software. Two-tailed unpaired Student’s *t* tests were conducted to evaluate the differences between the control and experimental groups. Significance is indicated by the presence of an asterisk (*p* < 0.05 is indicated by “*”; *p* < 0.01 is indicated by “**”;* p* < 0.001 is indicated by “***”).

## Results

### Detection of antigen-presenting ability, cell migration and phagocytosis of activated microglia

**Figure 1:**
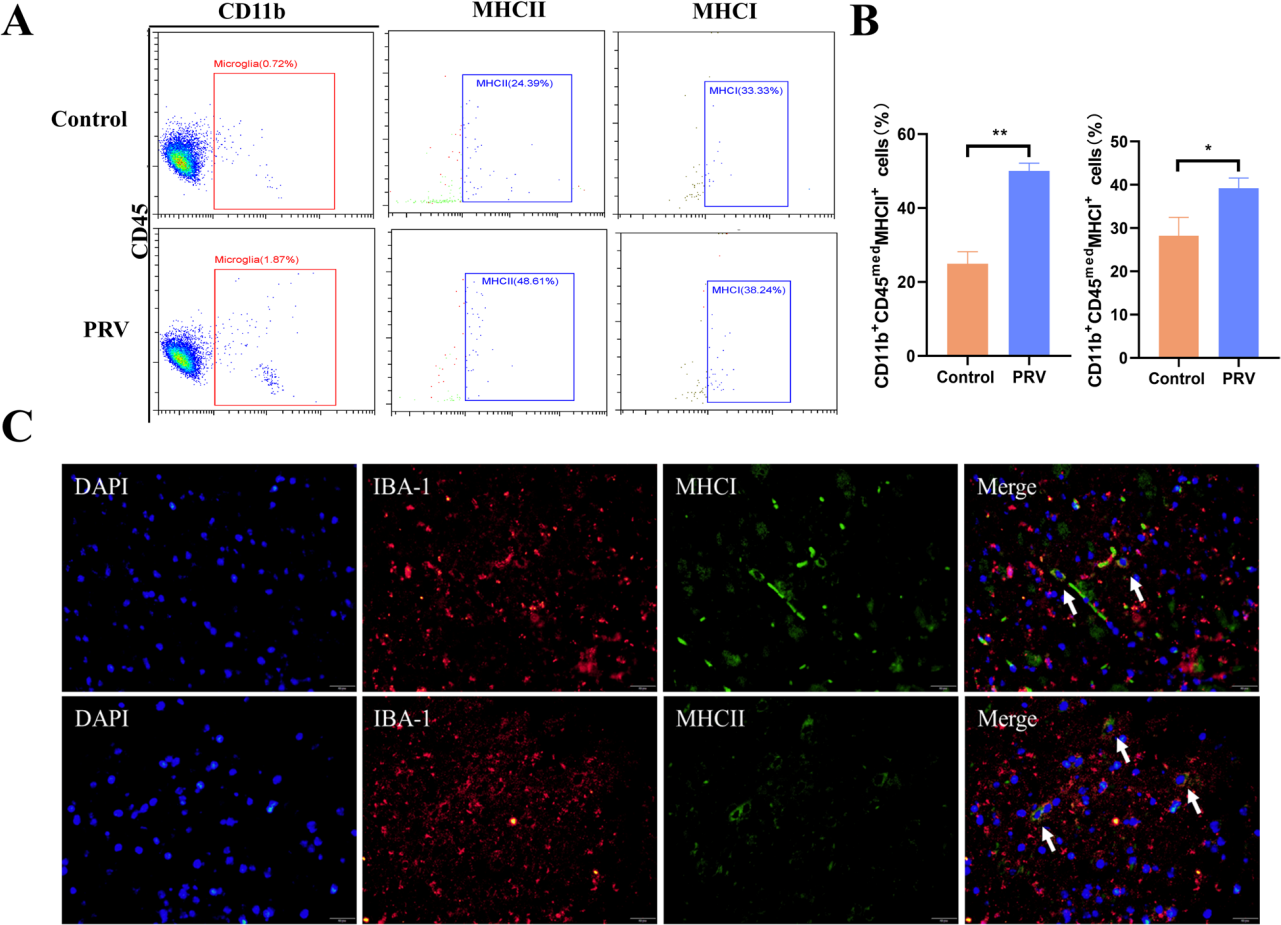
**Microglial antigen presentation**. **A** Mice (*n* = 6) were intranasally infected with PRV at 10^4^ TCID_50_, and single cells were isolated from the brain for flow cytometry analysis on day 4 post infection. Flow cytometry dot plot showing the relative expression of MHC I and MHC II in microglia (CD45^low^/CD11b^+^). **B** Positive rate of microglia expressing MHC. **C** Representative images showing the colocalization of Iba-1 (red), MHCI/MHCII (green), and DAPI (blue) in brain tissue via immunofluorescence (magnification, × 100).

Compared with those in mock-infected mice, a greater percentage of microglia in PRV-infected mice expressed MHCI (77.87%) and MHCII (88.89%) (Figures 1A and B). In addition, MHC positivity was confirmed by immunostaining, with arrows indicating colocalization of the Iba-1 signal with MHCI/MHCII in microglia (Figure 1C). The above described results show that activated microglia mediate inflammation through antigen presentation.

**Figure 2:**
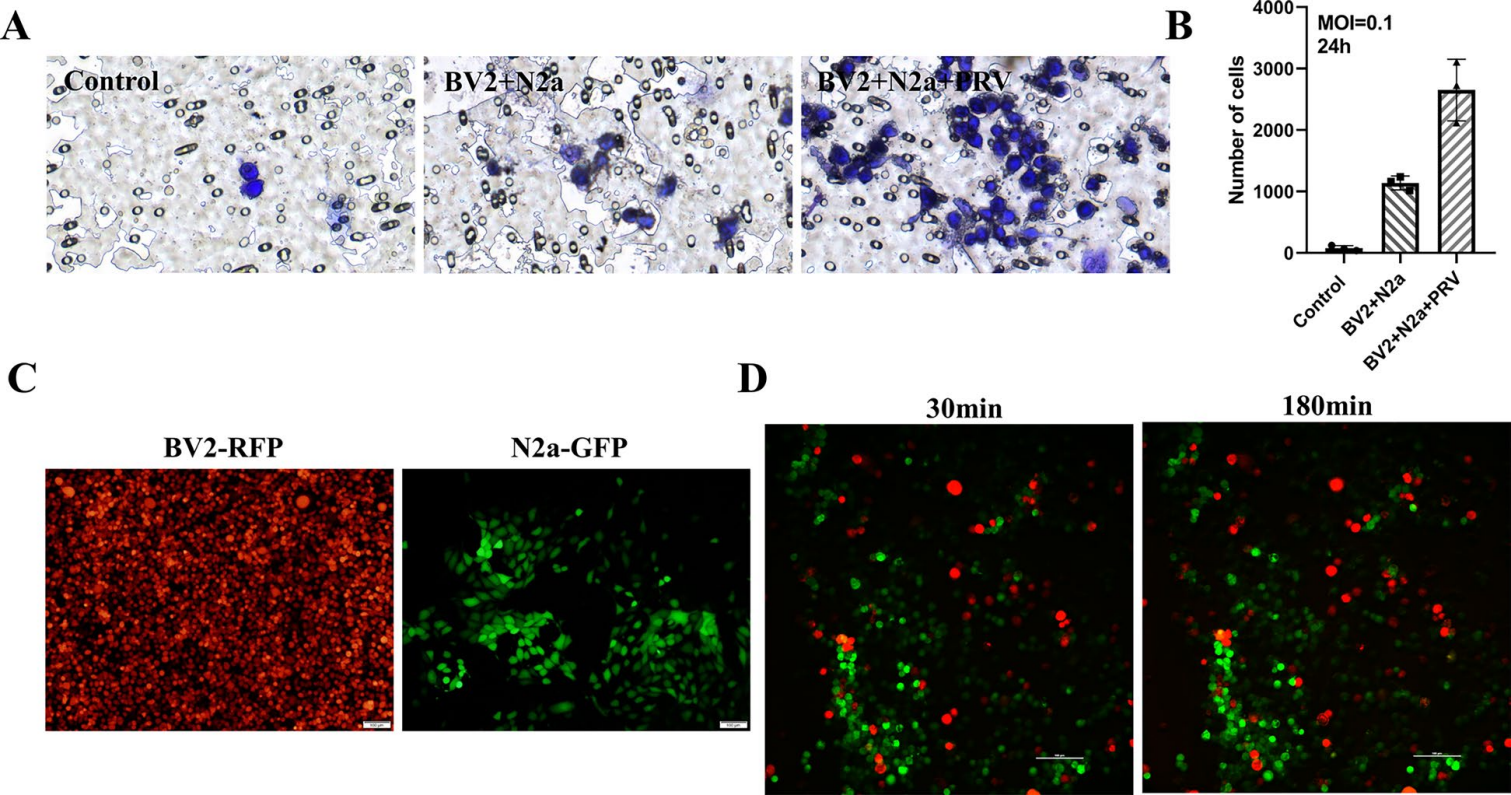
**Microglial migration and phagocytosis**. **A** Representative images showing BV2 migration in the Transwell coculture system (Corning, pore size = 0.4 µm) with or without N2a cell damage. **B** Quantification of migrating BV2 cells in the coculture system with or without N2a cell damage (*n* = 3). **C** Cells labelled with fluorescent proteins. **D** Representative images showing phagocytosis by BV2 cells in the coculture system with N2a cell damage.

In addition, a microglial migration assay demonstrated increased BV2 migration across Transwell membranes when N2a cells were infected with PRV (Figures 2A and B). For this purpose, we established BV2 cells expressing red fluorescent protein (RFP) and N2a cells expressing green fluorescent protein (GFP) (Figure 2C). PRV inoculation occurred after a 12-h coculture between these cell types. We observed BV2-RFP cell division and migration via a live cell workstation. BV2-RFP cells were observed to engulf N2a-GFP within 180 min (Figure 2D). The above described results show that activated microglia mediate inflammation through migration and phagocytosis.

### Detection of proinflammatory cytokines in the brain tissue of mice infected with PRV

**Figure 3:**
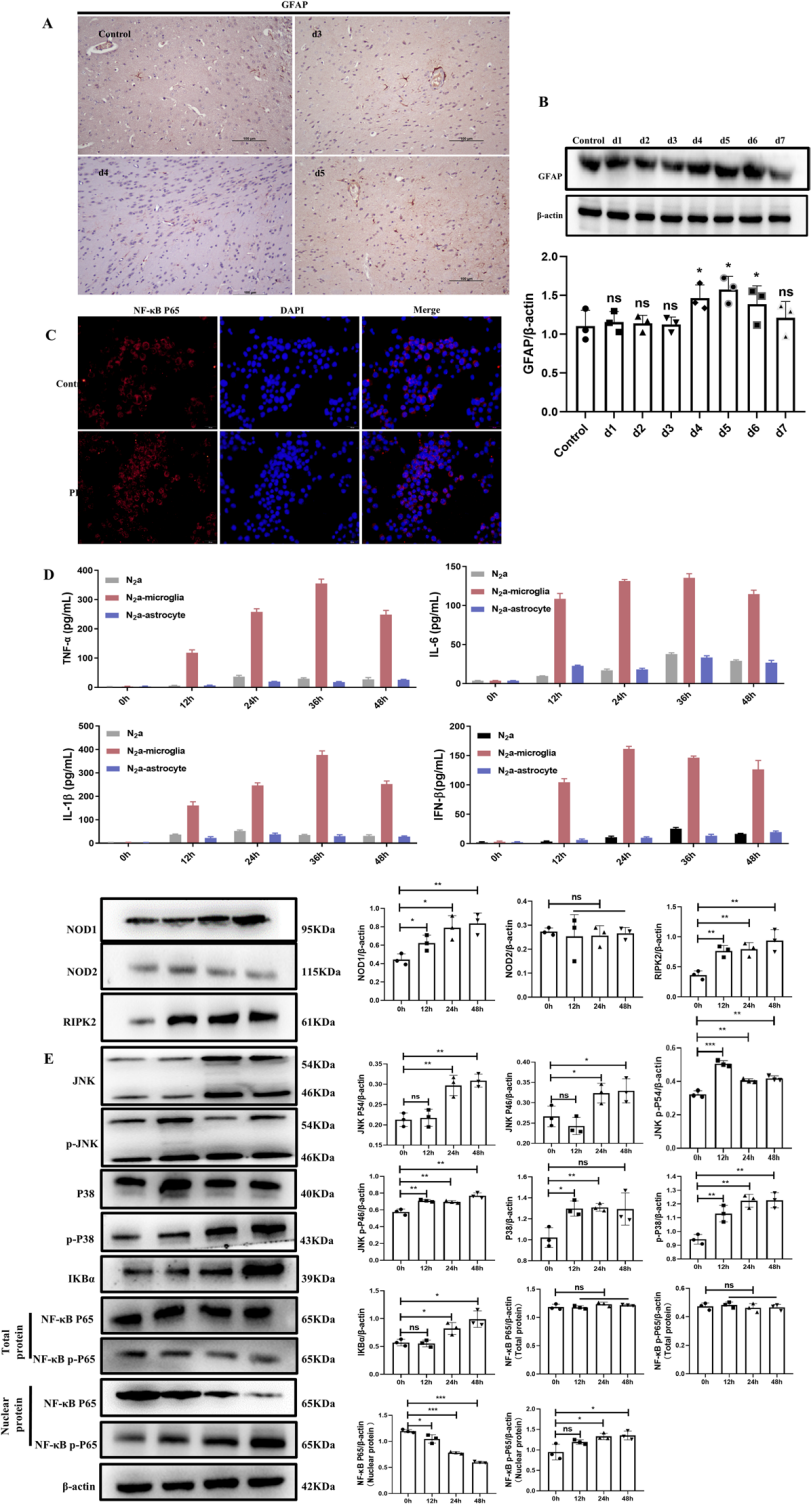
**Activation characteristics of microglia**. On days 3, 4, and 5 post-infection, brain tissue was collected aseptically from mice (intranasal infection with 10^4^ TCID_50_ PRV) after euthanasia. **A** Expression of GFAP in the brain tissues of mice determined by immunohistochemical staining (magnification, × 20). **B** Protein levels of GFAP and β-actin in the brain tissues of the mice were determined by western blotting. Primary microglia and astrocytes were isolated from primary mixed glial cells from 2-day-old mice. **C** Expression of NF-κB in BV2 cells infected with PRV. **D** TNF-α, IL-6, IL-1β, and IFN-β levels in the cell supernatant were detected via ELISA. **E** PRV induced the activation of BV2-related signalling pathways.

Microglia, the principal immune cells in the brain, play crucial roles in regulating immune responses and brain function. We observed the activation of astrocytes (Figures 3A and B). To confirm this further, we isolated primary astrocytes and microglia and established a neuron with a microglia/astrocyte indirect coculture model via a Transwell system. The lower layer neurons were infected with PRV. After 24 h, we detected inflammatory factors in the supernatants of the upper and lower cells. The results revealed that the levels of inflammatory factors secreted by microglia were significantly greater than those secreted by neurons and astrocytes (Figure 3D).

### Effect of PRV infection on the NOD1-NF-κB/MAPK signalling pathway in BV2 cells

IF was used to detect whether NF-κB entered the nucleus of BV2 cells at 48 h after PRV infection. As shown in Figure 3C, NF-κB was expressed mainly in the cytoplasm of the BV2 cells in the control group, and NF-κB was expressed mostly in the nucleus of the BV2 cells in the PRV infection group. IKBα dissociates and is degraded from the NF-κB complex, exposing the nuclear localization sequence of NF-κB. NF-κB undergoes nuclear translocation, enters the nucleus and binds to specific sequences. These results indicate that the NOD1-NF-κB/MAPK pathway is involved in the activation of BV2 cells induced by PRV.

To elucidate the signalling pathways underlying the activation of microglia during PRV infection, we employed transcriptome sequencing to identify differentially expressed mRNAs in PRV-infected BV2 cells. The results of transcriptome sequencing revealed that the activation of BV2 cells induced by PRV infection triggered an inflammatory response, promoted the secretion of many inflammatory cytokines, and activated related signalling pathways. Therefore, the related signalling pathways with significant differences in KEGG enrichment were verified. BV2 cells were infected with PRV at an MOI of 1, and then samples were collected at different times for detection. As shown in Figure 3E, the expression of NOD1, a member of the nucleotide-binding oligomerization structural protein-like receptor (NLR) family, was significantly upregulated at 12 h of infection in a time-dependent manner, whereas there was no difference in NOD2 expression. The downstream protein RIPK2 and the related signalling pathways NF-κB and MAPK were subsequently detected. The results revealed that the expression of RIPK2 increased at 12 h after infection in a time-dependent manner. The expression of the key proteins JNK, p-JNK, P38 and p-P38 in the MAPK pathway increased at 12 h after infection in a time-dependent manner. The expression of IKBα in the NF-κB pathway was increased, and the expression of NF-κB P65 in the nucleus was decreased, while the expression of NF-κB P65 in the total protein was not detected.

### Effect of NOD1 silencing on BV2 cells during PRV infection

**Figure 4:**
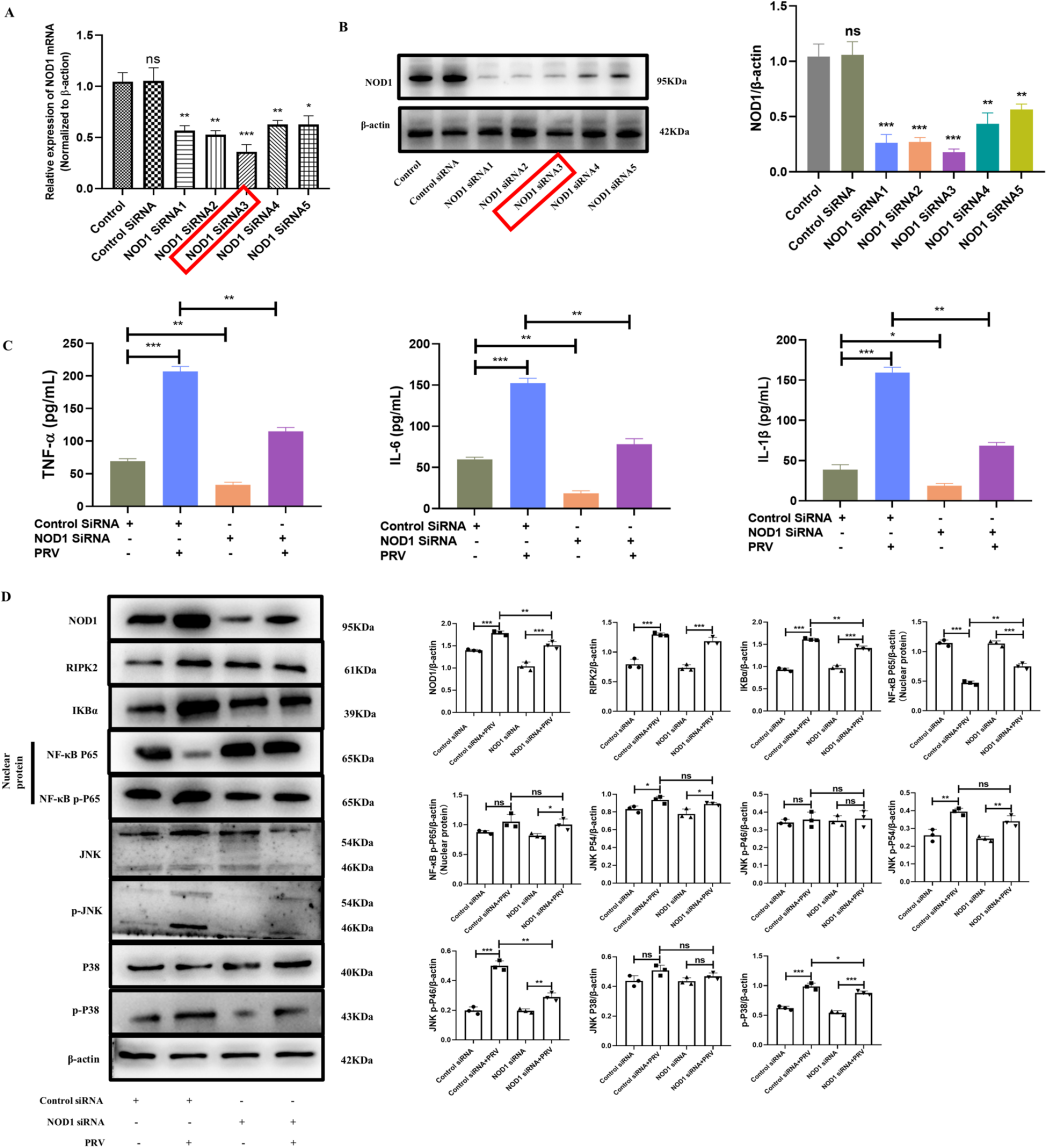
**Effect of NOD1 silencing on BV2 cells during PRV infection**. **A** Expression of NOD1 in BV2 cells at the mRNA level by qRT‒PCR; **B** expression of NOD1 in BV2 cells at the protein level by WB. **C** Effect of NOD1 silencing on inflammatory cytokine expression in BV2 cells during PRV infection. **D** Effect of NOD1 silencing on the NF-κB/MAPK pathway in BV2 cells during PRV infection.

To study the role of NOD1 in the PRV-induced inflammatory response in BV2 cells, siRNA was used to silence the NOD1 gene and inhibit NOD1 mRNA expression. After the transfection of BV2 cells with NOD1 siRNA, the transfection efficiency was detected at the mRNA and protein levels. The expression level of NOD1 was detected at the mRNA level at 24 h after transfection. The results are shown in Figure 4A. Compared with that in the control siRNA group, the expression level of NOD1 in the 5 pairs of siRNAs was lower (*p* < 0.001). The expression level of NOD1 was detected at 48 h after transfection. The results are shown in Figure 4B. Compared with that in the control siRNA group, the expression level of NOD1 in the 5 pairs of siRNAs was lower (*p* < 0.001). Therefore, NOD1 siRNA3 was selected for subsequent experiments.

ELISA was used to detect the expression of inflammatory cytokines in the supernatants of BV2 cells transfected with NOD1 siRNA for 48 h. Compared with control siRNA, PRV infection significantly increased the expression of TNF-α, IL-6 and IL-1β (*p* < 0.001). Compared with the control siRNA, NOD1 gene silencing significantly inhibited the expression of TNF-α (p < 0.01), IL-6 (*p* < 0.01) and IL-1β (*p* < 0.05). Compared with PRV infection alone, NOD1 gene silencing significantly inhibited the expression of TNF-α, IL-6 and IL-1β induced by PRV (*p* < 0.01) (Figure 4C).

To study the effect of NOD1 on the NF-κB/MAPK pathway in BV2 cells during PRV infection, siRNA was used to silence NOD1, and the expression of RIPK2, IKBα, NF-κB P65, NF-κB p-P65, JNK, p-JNK, P38 and p-P38 in BV2 cells was detected at 48 h. The transfected BV2 cells were infected with PRV at an MOI of 1. The results are shown in Figure 4D. The expression of NOD1, RIPK2, IKBα, NF-κB P65, NF-κB p-P65, JNK, p-JNK, P38 and p-P38 in the NOD siRNA + PRV transfection group was significantly lower than that in the control siRNA + PRV group.

### Effect of NOD1 overexpression on BV2 cells during PRV infection

**Figure 5:**
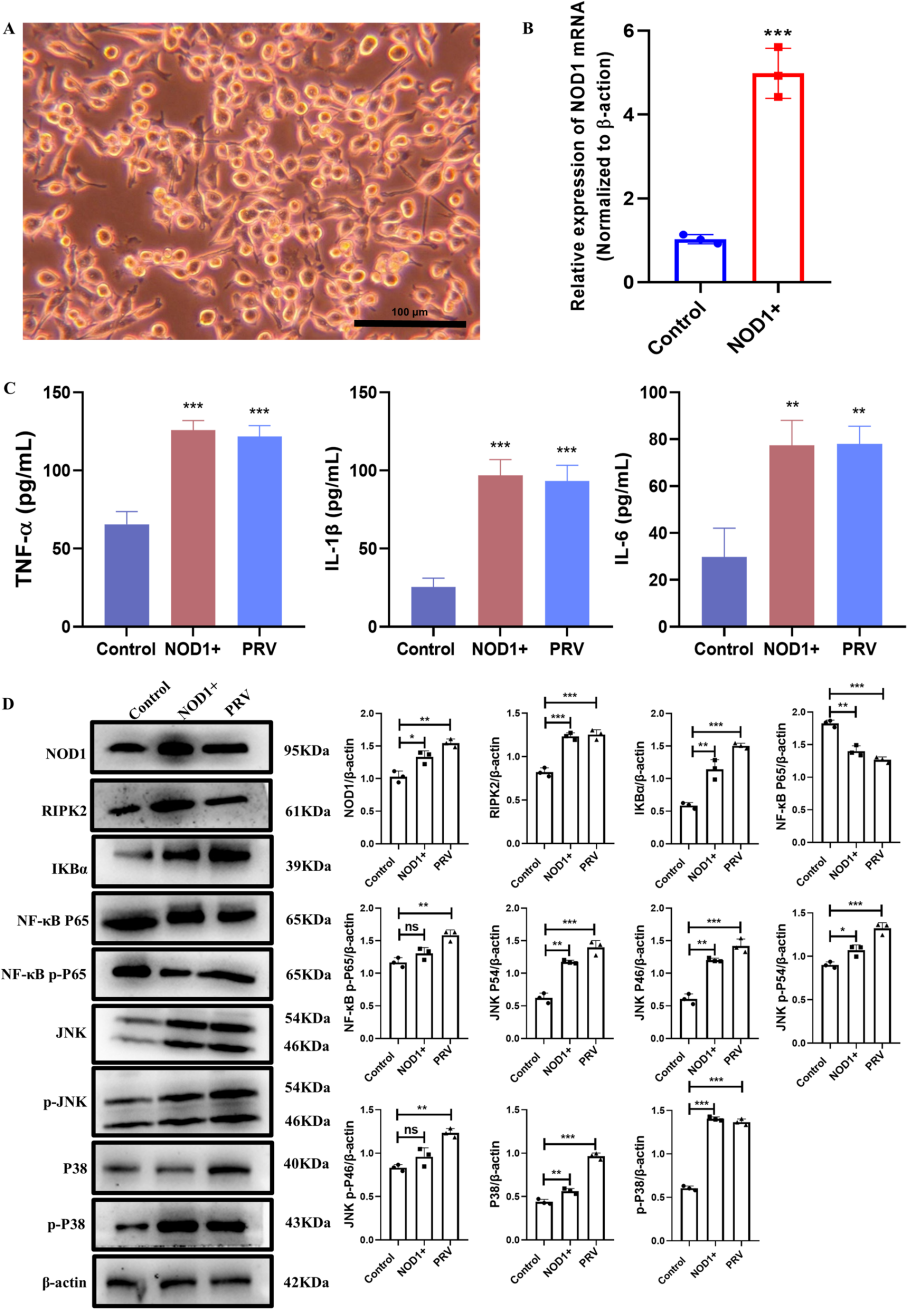
**Effects of NOD1 overexpression on BV2 cells**. **A** Expression of RFP in BV2 cells under a microscope; **B** expression of NOD1 in BV2 cells at the mRNA level by qRT‒PCR. **C** Effects of NOD1 overexpression on inflammatory cytokine expression in BV2 cells during PRV infection. **D** Effect of NOD1 overexpression on the NF-κB/MAPK pathway in BV2 cells during PRV infection.

The NOD1 plasmid was constructed and then transfected into BV2 cells to achieve NOD1 overexpression in BV2 cells. After the transfection of the NOD1 plasmid, the growth of the BV2 cells was observed under a microscope. As shown in Figure 5A, BV2 cells grew well at 24 h after transfection with the NOD1 plasmid. The transfection efficiency of NOD1 was detected by qRT‒PCR. The results are shown in Figure 5B. Compared with that in the empty vector group, the expression of NOD1 mRNA in BV2 cells was significantly increased after NOD1 plasmid transfection (*p* < 0.001). The expression levels of inflammatory cytokines in the cell supernatant were detected at 24 h after transfection. As shown in Figure 5C, compared with those in the control group, the levels of TNF-α, IL-6 and IL-1β in the supernatants of BV2 cells were significantly increased at 24 h after PRV infection (*p* < 0.01), and the levels of TNF-α, IL-6 and IL-1β in the supernatants of BV2 cells after NOD1 overexpression were also significantly increased (*p* < 0.01). The expression of RIPK2, IKBα, NF-κB P65, NF-κB p-P65, JNK, p-JNK, P38 and p-P38 in BV2 cells was detected at 24 h after transfection. As shown in Figure 5D, the expression of RIPK2, IKBα, NF-κB P65, NF-κB p-P65, JNK, p-JNK, P38 and p-P38 in BV2 cells was significantly greater than that in the control group at 24 h after NOD1 plasmid transfection, which was consistent with the trend observed in the PRV infection group.

### Effect of PRV infection on the NOD1-NF-κB/MAPK pathway in mouse brain tissue

**Figure 6:**
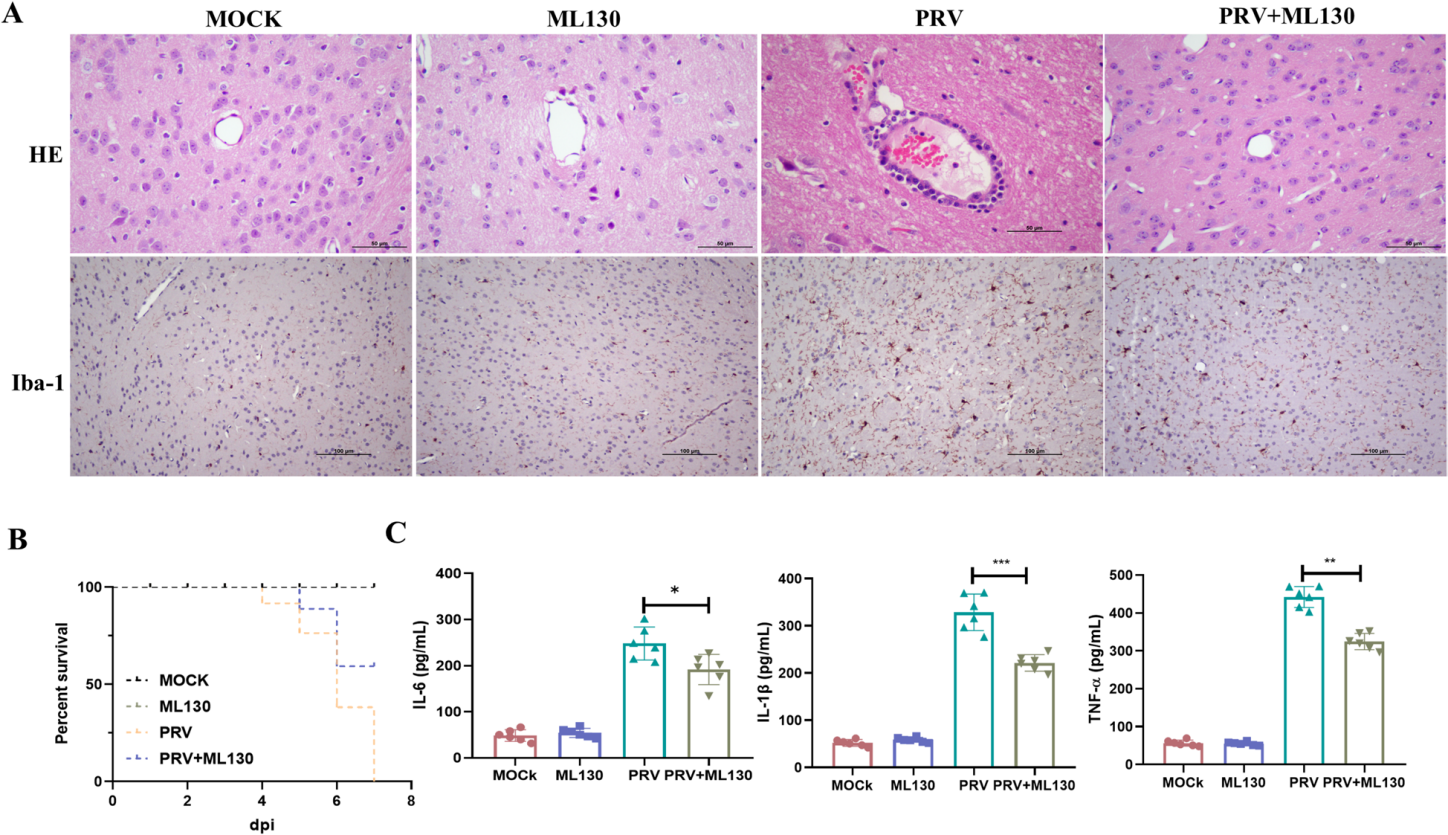
**Effects of PRV infection on the NOD1-NF-κB/MAPK pathway in mouse brain tissue**. **A** HE staining and immunohistochemistry were used to observe pathological changes and activation of microglia. **B** Survival rate of the mice. **C** ELISA detection of TNF-α, IL-6 and IL-1β expression in the brain tissue of the mice.

Inflammation is an important pathological manifestation of encephalitis and is caused mainly by glial cell activation and inflammatory factor secretion. To evaluate the role of NOD1 in VE caused by PRV infection, ML130 was injected into the lateral ventricles of the mice to inhibit the expression of NOD1, after which they were infected with PRV. As shown in Figure 6A, compared with those in the mice in the PRV group, the pathological changes in the brain tissue in the PRV + ML130 group were significantly reduced (peripheral immune cell infiltration was reduced, apoptosis was reduced), and the number of activated microglia in the brain tissue was reduced. The survival rate of the mice in each group was calculated. The results revealed that the survival rate of the mice in the PRV + ML130 group was greater than that in the PRV infection group (Figure 6B). In addition, the secretion of inflammatory cytokines in the brain tissue of mice infected with ML130 was reduced (Figure 6C).

**Figure 7:**
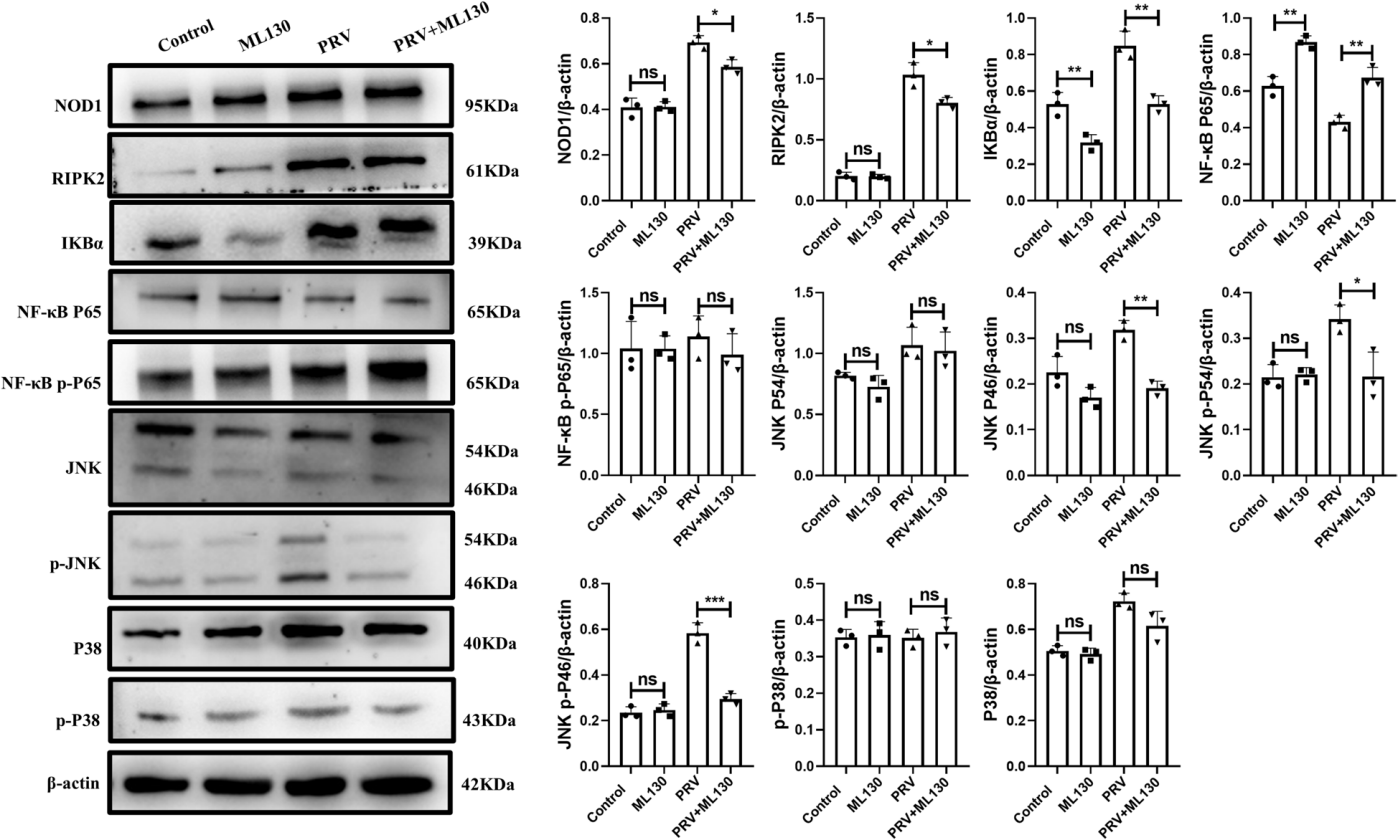
**Inhibition of NOD1 reduces the expression of the NF-κB/MAPK pathway in mouse brain tissues infected with PRV**.

To determine the NOD1 signalling pathway involved in VE caused by PRV infection, the expression of molecules downstream of NOD1 signalling in mouse brain tissue was detected. As shown in Figure 7, ML130 significantly affected the expression of RIPK2, IKBα, NF-κB P65, NF-κB p-P65, JNK, p-JNK, P38 and p-P38. The expression of RIPK2, IKBα, NF-κB P65, NF-κB p-P65, JNK, p-JNK, P38 and p-P38 in the PRV + ML130 group was significantly lower than that in the PRV group.

### Effect of PRV on the NOD1-NF-κB/MAPK pathway in pig brain tissue

**Figure 8:**
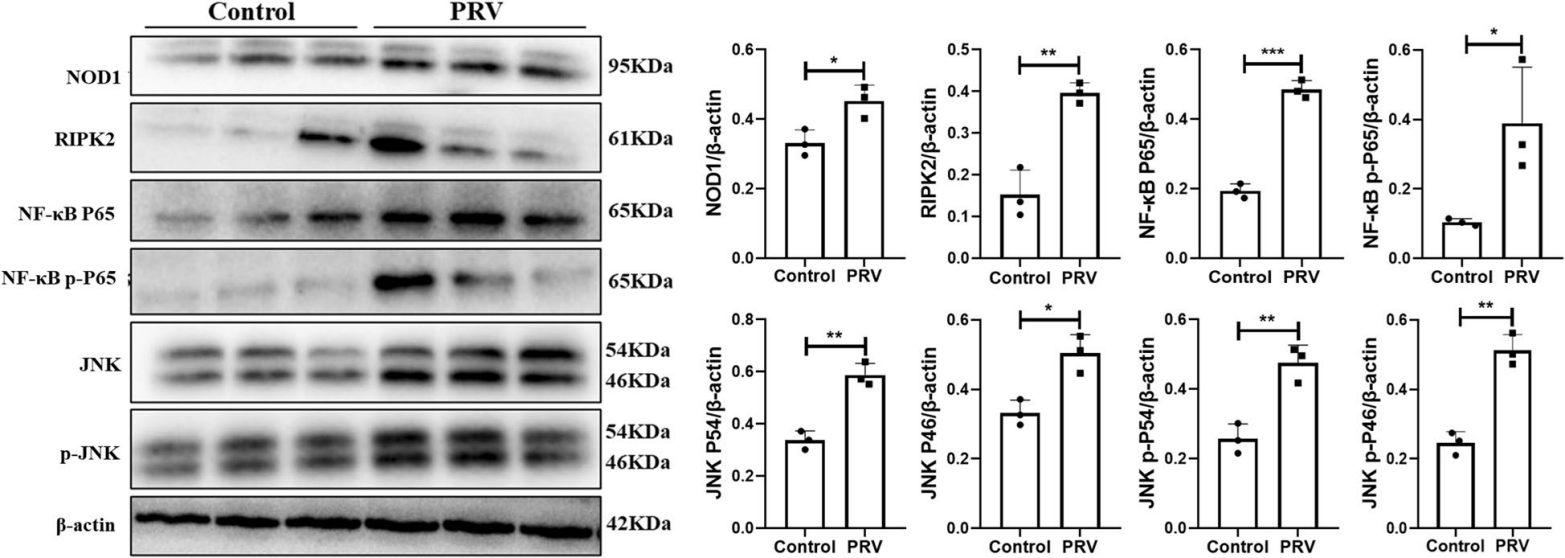
**Expression of the NF-κB/MAPK pathway in pig brain tissues**.

Both cell and mouse experiments revealed that NOD1-NF-κB/MAPK is involved in regulating the secretion of inflammatory factors in microglia. To further verify whether the results can be applied in pigs, the expression of the NOD1-NF-κB/MAPK pathway in pig brain tissue after PRV infection was detected at the protein level. As shown in Figure 8, the expression of NOD1, RIPK2, NF-κB, p-NF-κB, JNK and p-JNK in the brain tissue of pigs infected with PRV was significantly increased.

## Discussion

One function of microglia is to initiate antigen-specific T-cell responses by presenting antigens via MHC molecules [27]. Microglia were isolated from the brains of PRV-infected mice, and the activation profiles of MHC I and MHC II were analysed by flow cytometry. Microglia, the resident immune cells in the brain, provide immune surveillance [28]. As resident immune cells in the CNS, activated microglia eliminate damaged neurons and pathogens in response to CNS stimulation or damage [27, 28]. During invasion, microglia are activated rapidly, undergo transformation to an amoeboid-like morphology, migrate to the site of pathology, release cytokines, execute phagocytosis activity, and engage in antigen presentation to maintain CNS homeostasis [27, 29–33]. Previous studies have shown rapid activation and polarization of microglia in PRV-infected mice, leading to the secretion of cytokines/chemokines to recruit immune cells [34]. In the present study, we provide the first evidence of microglial activation by PRV infection. Microglia, as resident immune cells, are the major producers of inflammatory mediators [35–37]. This is also reflected in our results. However, microglia play dual roles in the CNS. Overactivated microglia release cytotoxins in the CNS, leading to neuronal death [38]. Therefore, we hypothesize that appropriately modulating microglial activation may provide a potential target for alleviating the symptoms of neuroinflammation.

NOD1 and NOD2 are well-characterized contributors to human disease. Evidence shows that NOD1 exerts its restrictive role by altering macrophage polarization in induced gastric cancer, leading to immune evasion and microbial persistence [39–42].

Moreover, increased NOD1 expression is linked to reduced overall survival in individuals with colorectal cancer [43–46]. NOD1 is widely expressed across various tissues [47]. Originally identified as inducers of the NF-κB pathway in response to bacterial pathogens by recognizing peptidoglycans within the bacterial cell wall, both NOD1 and NOD2 receptors play a role in this process [43, 48–50]. RIPK2 serves as a crucial downstream adaptor for both NOD1 and NOD2, playing an essential role in activating NF-κB signalling [51–54]. However, the role of NOD1/RIPK2 in the brain remains unclear. This study reveals, for the first time, that NOD1/RIPK2 signalling, rather than NOD2 signalling, contributes to brain damage and positively regulates neuroinflammation induced by PRV infection by attenuating the NF-κB and JNK signalling pathways in mice. Notably, NOD1 and NOD2 are involved in the brain tissue of PRV-infected pigs.

Some studies have demonstrated that NOD1 can be expressed in leukocytes, including monocytes/macrophages, DCs, stromal cells, hematopoietic cells, and epithelial cells, upon inflammatory stimulation [55–57]. Our results demonstrated that NOD1 was expressed in microglia. Inflammatory cytokine expression was significantly inhibited after knockdown of the NOD1 gene in microglia. This finding is consistent with the results of the animal experiments. Therefore, NOD1 is likely involved in PRV-induced neuroinflammation by regulating the production and secretion of inflammatory cytokines in activated microglia.

**Figure 9:**
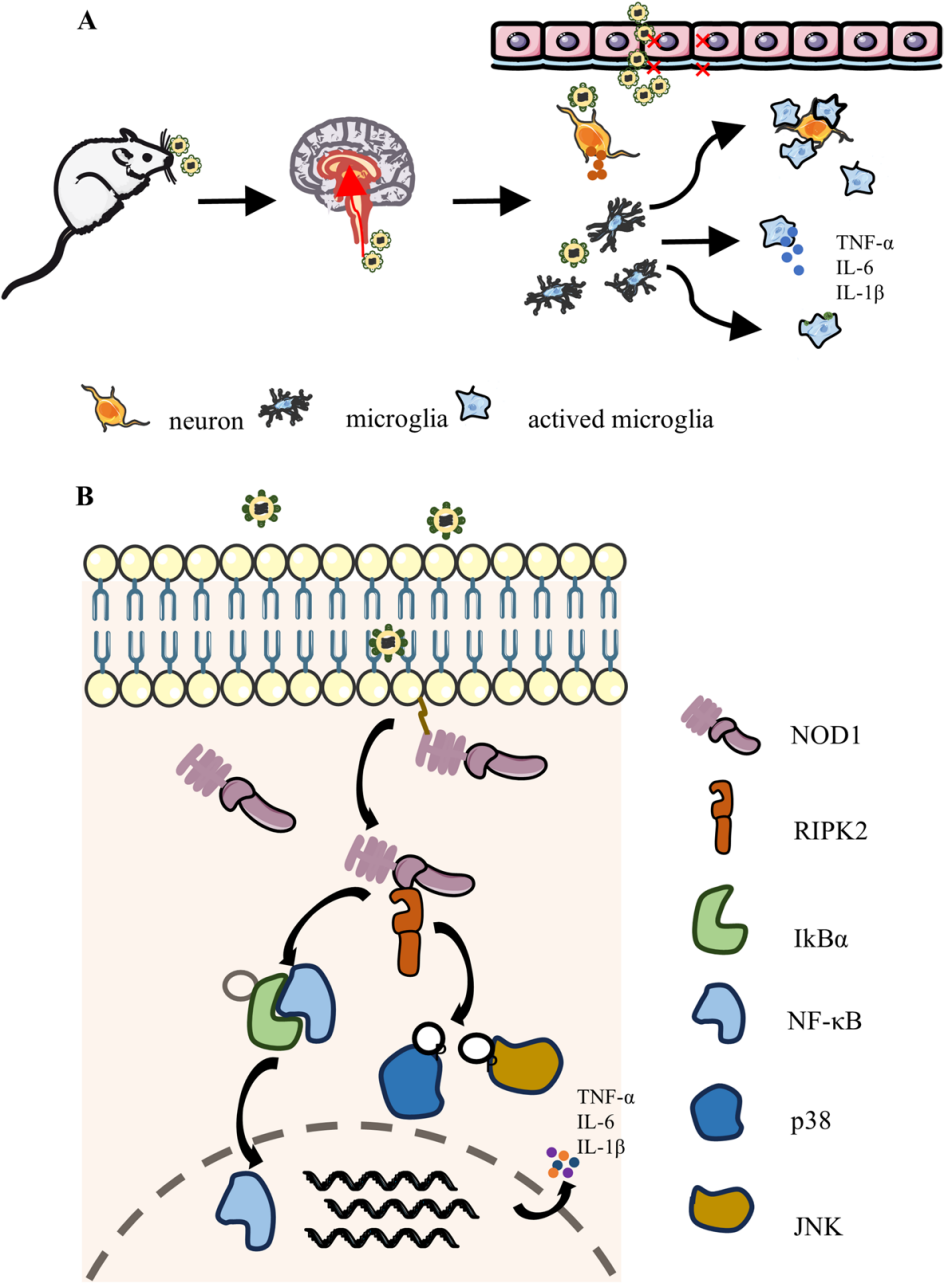
**Proposed mechanism for the pathogenicity of NOD1/RIPK2 signalling in mediating microglial activation and the inflammatory response**. **A** PRV can infiltrate the brain through the periphery of mice via intranasal PRV infection. Microglia sense changes in the surrounding environment and are promptly activated. The activation of microglia involves morphological changes, migration, release of inflammatory factors, and antigen presentation. **B** In the presence of PRV, NOD1 is activated, leading to the activation of its adaptor, RIPK2. RIPK2 regulates the proinflammatory factors TNF-α, IL-6, and IL-1β by modulating the JNK and NF-κB signalling pathways.

The inhibition of NOD1 diminishes neuropathogenesis and safeguards mice from lethality during PRV infection. In conclusion, for the first time, we demonstrated that NOD1 may mitigate PRV-induced neuroinflammation by regulating microglia-mediated responses (Figure 9). Therefore, targeting NOD1 is a potential therapeutic approach for addressing PRV pathogenesis.

## Data Availability

The authors confirm that the data supporting the findings of this study are available within the article and its additional files. The data that support the findings of this study are available from the corresponding author upon reasonable request.

## References

[1] McGeoch DJ, Cook S (1994) Molecular phylogeny of the alphaherpesvirinae subfamily and a proposed evolutionary timescale. J Mol Biol 238:9–22 8145260 10.1006/jmbi.1994.1264

[2] Pomeranz LE, Reynolds AE, Hengartner CJ (2005) Molecular biology of pseudorabies virus: impact on neurovirology and veterinary medicine. Microbiol Mol Biol Rev 69:462–500 16148307 10.1128/MMBR.69.3.462-500.2005 PMC1197806

[3] Fooks AR, Cliquet F, Finke S, Freuling C, Hemachudha T, Mani RS (2017) Rabies. Nat Rev Dis Primers 3:17091 29188797 10.1038/nrdp.2017.91

[4] Reinert LS, Lopušná K, Winther H, Sun C, Thomsen MK, Nandakumar R, Mogensen TH, Meyer M, Vægter C, Nyengaard JR, Fitzgerald KA, Paludan SR (2016) Sensing of HSV-1 by the cGAS-STING pathway in microglia orchestrates antiviral defence in the CNS. Nat Commun 7:13348 27830700 10.1038/ncomms13348 PMC5109551

[5] He W, Auclert LZ, Zhai X, Wong G, Zhang C, Zhu H, Xing G, Wang S, He W, Li K, Wang L, Han GZ, Veit M, Zhou J, Su S (2019) Interspecies transmission, genetic diversity, and evolutionary dynamics of pseudorabies virus. J Infect Dis 219:1705–1715 30590733 10.1093/infdis/jiy731

[6] Card JP, Rinaman L, Schwaber JS, Miselis RR, Whealy ME, Robbins AK, Enquist LW (1990) Neurotropic properties of pseudorabies virus: uptake and transneuronal passage in the rat central nervous system. J Neurosci 10:1974–1994 2162388 10.1523/JNEUROSCI.10-06-01974.1990 PMC6570305

[7] Card JP, Rinaman L, Schwaber JS, Miselis RR, Whealy ME, Robbins AK, Enquist LW (1990) Human encephalitis caused by pseudorabies virus infection: a case report. J Neurovirol 26:442–448 10.1007/s13365-019-00822-2 PMC7223082 31898060

[8] Yang X, Guan H, Li C, Li Y, Wang S, Zhao X, Zhao Y, Liu Y (2019) Characteristics of human encephalitis caused by pseudorabies virus: a case series study. Int J Infect Dis 87:92–99 31408708 10.1016/j.ijid.2019.08.007

[9] Wong G, Lu J, Zhang W, Gao GF (2019) Pseudorabies virus: a neglected zoonotic pathogen in humans? Emerg Microbes Infect 8:150–154 30866769 10.1080/22221751.2018.1563459 PMC6455137

[10] Ai JW, Weng SS, Cheng Q, Cui P, Li YJ, Wu HL, Zhu YM, Xu B, Zhang WH (2018) Human endophthalmitis caused by pseudorabies virus infection. Emerg Infect Dis 24:1087–1090 29774834 10.3201/eid2406.171612 PMC6004832

[11] Wang J (2010) Preclinical and clinical research on inflammation after intracerebral hemorrhage. Prog Neurobiol 92:463–477 20713126 10.1016/j.pneurobio.2010.08.001 PMC2991407

[12] Graeber MB (2010) Changing face of microglia. Science 330:783–788 21051630 10.1126/science.1190929

[13] Dheen ST, Kaur C, Ling EA (2007) Microglial activation and its implications in the brain diseases. Curr Med Chem 14:1189–1197 17504139 10.2174/092986707780597961

[14] Cymerys J, Kowalczyk A, Mikołajewicz K, Słońska A, Krzyżowska M (2019) Nitric oxide influences HSV-1-induced neuroinflammation. Oxid Med Cell Longev 2019:2302835 30886672 10.1155/2019/2302835 PMC6388346

[15] Leyshon BJ, Ji P, Caputo MP, Matt SM, Johnson RW (2019) Dietary iron deficiency impaired peripheral immunity but did not alter brain microglia in PRRSV-infected neonatal piglets. Front Immunol 9:3150 30778359 10.3389/fimmu.2018.03150 PMC6369153

[16] Kim DH, Yoon BH, Jung WY, Kim JM, Park SJ, Park DH, Huh Y, Park C, Cheong JH, Lee KT, Shin CY, Ryu JH (2010) Sinapic acid attenuates kainic acid-induced hippocampal neuronal damage in mice. Neuropharmacology 59:20–30 20363233 10.1016/j.neuropharm.2010.03.012

[17] Correa RG, Milutinovic S, Reed JC (2012) Roles of NOD1 (NLRC1) and NOD2 (NLRC2) in innate immunity and inflammatory diseases. Biosci Rep 32:597–608 22908883 10.1042/BSR20120055 PMC3497720

[18] Kawai T, Akira S (2011) Toll-like receptors and their crosstalk with other innate receptors in infection and immunity. Immunity 34:637–650 21616434 10.1016/j.immuni.2011.05.006

[19] Chamaillard M, Hashimoto M, Horie Y, Masumoto J, Qiu S, Saab L, Ogura Y, Kawasaki A, Fukase K, Kusumoto S, Valvano MA, Foster SJ, Mak TW, Nuñez G, Inohara N (2003) An essential role for NOD1 in host recognition of bacterial peptidoglycan containing diaminopimelic acid. Nat Immunol 4:702–707 12796777 10.1038/ni945

[20] Chen Z, Zhao Z, Liu Y, Imran M, Rao J, Cai N, Ye J, Cao S (2022) Nucleotide-binding oligomerization domain 1 (NOD1) positively regulates neuroinflammation during Japanese encephalitis virus infection. Microbiol Spectr 10:e0258321 35638852 10.1128/spectrum.02583-21 PMC9241932

[21] Mukherjee T, Hovingh ES, Foerster EG, Abdel-Nour M, Philpott DJ, Girardin SE (2018) NOD1 and NOD2 in inflammation, immunity and disease. Arch Biochem Biophys 670:69–81 30578751 10.1016/j.abb.2018.12.022

[22] Fan YH, Roy S, Mukhopadhyay R, Kapoor A, Duggal P, Wojcik GL, Pass RF, Arav-Boger R (2016) Role of nucleotide-binding oligomerization domain 1 (NOD1) and its variants in human cytomegalovirus control in vitro and in vivo. Proc Natl Acad Sci USA 113:7818–7827 10.1073/pnas.1611711113 PMC5137695 27856764

[23] Magalhaes JG, Lee J, Geddes K, Rubino S, Philpott DJ, Girardin SE (2011) Essential role of Rip2 in the modulation of innate and adaptive immunity triggered by Nod1 and Nod2 ligands. Eur J Immunol 41:1445–1455 21469090 10.1002/eji.201040827

[24] Guo XX, Li XP, Zhou P, Li DY, Lyu XT, Chen Y, Lyu YW, Tian K, Yuan DZ, Ran JH, Chen DL, Jiang R, Li J (2018) Evodiamine induces apoptosis in SMMC-7721 and HepG2 cells by suppressing NOD1 signal pathway. Int J Mol Sci 19:3419 30384473 10.3390/ijms19113419 PMC6274686

[25] Gall A, Gaudet RG, Gray-Owen SD, Salama NR (2017) TIFA signaling in gastric epithelial cells initiates the *cag* type 4 secretion system-dependent innate immune response to *Helicobacter pylori* infection. MBio 8:e01168–17 28811347 10.1128/mBio.01168-17 PMC5559637

[26] Chen H, Fan J, Sun X, Xie R, Song W, Zhao Y, Yang T, Cao Y, Yu S, Wei C, Hua L, Wang X, Chen H, Peng Z, Cheng G, Wu B (2023) Characterization of pseudorabies virus associated with severe respiratory and neuronal signs in old pigs. Transbound Emerg Dis 2023:855739 10.1155/2023/8855739 PMC12017139 40303768

[27] Mack CL, Vanderlugt-Castaneda CL, Neville KL, Miller SD (2003) Microglia are activated to become competent antigen presenting and effector cells in the inflammatory environment of the Theiler’s virus model of multiple sclerosis. J Neuroimmunol 144:68–79 14597100 10.1016/j.jneuroim.2003.08.032

[28] Dong H, Wang Y, Zhang X, Zhang X, Qian Y, Ding H, Zhang S (2019) Stabilization of brain mast cells alleviates LPS-induced neuroinflammation by inhibiting microglia activation. Front Cell Neurosci 13:191 31130850 10.3389/fncel.2019.00191 PMC6509474

[29] Hammond TR, Dufort C, Dissing-Olesen L, Giera S, Young A, Wysoker A, Walker AJ, Gergits F, Segel M, Nemesh J, Marsh SE, Saunders A, Macosko E, Ginhoux F, Chen J, Franklin RJM, Piao X, McCarroll SA, Stevens B (2019) Single-cell RNA sequencing of microglia throughout the mouse lifespan and in the injured brain reveals complex cell-state changes. Immunity 50:253–271 30471926 10.1016/j.immuni.2018.11.004 PMC6655561

[30] Salter MW, Stevens B (2017) Microglia emerge as central players in brain disease. Nat Med 23:1018–1027 28886007 10.1038/nm.4397

[31] Fu R, Shen Q, Xu P, Luo JJ, Tang Y (2014) Phagocytosis of microglia in the central nervous system diseases. Mol Neurobiol 49:1422–1434 24395130 10.1007/s12035-013-8620-6 PMC4012154

[32] Hafizi S, Da Silva T, Meyer JH, Kiang M, Houle S, Remington G, Prce I, Wilson AA, Rusjan PM, Sailasuta N, Mizrahi R (2018) Interaction between TSPO-a neuroimmune marker-and redox status in clinical high risk for psychosis: a PET-MRS study. Neuropsychopharmacology 43:1700–1705 29748630 10.1038/s41386-018-0061-5 PMC6006145

[33] Ela S, Mäger I, Breakefield XO, Wood MJ (2013) Extracellular vesicles: biology and emerging therapeutic opportunities. Nat Rev Drug Discov 12:347–357 23584393 10.1038/nrd3978

[34] Sun X, Jin X, Liu X, Wang L, Li L, Yang J, Feng H, Lin Z, Zhan C, Zhang W, Gu C, Hu X, Liu X, Cheng G (2023) Microglia play an important role in PRV infection-induced immune responses of the central nervous system. Virol J 20:151 37452371 10.1186/s12985-023-02118-8 PMC10349424

[35] Heppner FL, Ransohoff RM, Becher B (2015) Immune attack: the role of inflammation in Alzheimer disease. Nat Rev Neurosci 16:358–372 25991443 10.1038/nrn3880

[36] Hanisch UK (2002) Microglia as a source and target of cytokines. Glia 40:140–155 12379902 10.1002/glia.10161

[37] Batista CRA, Gomes GF, Candelario-Jalil E, Fiebich BL, de Oliveira ACP (2019) Lipopolysaccharide-induced neuroinflammation as a bridge to understand neurodegeneration. Int J Mol Sci 20:2239 31075861 10.3390/ijms20092293 PMC6539529

[38] Stephenson J, Nutma E, van der Valk P, Amor S (2018) Inflammation in CNS neurodegenerative diseases. Immunology 154:204–219 29513402 10.1111/imm.12922 PMC5980185

[39] Suarez G, Romero-Gallo J, Piazuelo MB, Sierra JC, Delgado AG, Washington MK, Shah SC, Wilson KT, Peek RM Jr (2019) Nod1 imprints inflammatory and carcinogenic responses toward the gastric pathogen *Helicobacter pylori*. Cancer Res 79:1600–1611 30696658 10.1158/0008-5472.CAN-18-2651 PMC6445772

[40] Li ZX, Wang YM, Tang FB, Zhang L, Zhang Y, Ma JL, Zhou T, You WC, Pan KF (2015) NOD1 and NOD2 genetic variants in association with risk of gastric cancer and its precursors in a Chinese population. PLoS One 10:e0124949 25933107 10.1371/journal.pone.0124949 PMC4416772

[41] Allison CC, Ferrand J, McLeod L, Hassan M, Kaparakis-Liaskos M, Grubman A, Bhathal PS, Dev A, Sievert W, Jenkins BJ, Ferrero RL (2013) Nucleotide oligomerization domain 1 enhances IFN-γ signaling in gastric epithelial cells during *Helicobacter pylori* infection and exacerbates disease severity. J Immunol 190:3706–3715 23460743 10.4049/jimmunol.1200591

[42] Rosenstiel P, Hellmig S, Hampe J, Ott S, Till A, Fischbach W, Sahly H, Lucius R, Fölsch UR, Philpott D, Schreiber S (2006) Influence of polymorphisms in the NOD1/CARD4 and NOD2/CARD15 genes on the clinical outcome of *Helicobacter pylori* infection. Cell Microbiol 8:1188–1198 16819970 10.1111/j.1462-5822.2006.00701.x

[43] Jiang HY, Najmeh S, Martel G, MacFadden-Murphy E, Farias R, Savage P, Leone A, Roussel L, Cools-Lartigue J, Gowing S, Berube J, Giannias B, Bourdeau F, Chan CHF, Spicer JD, McClure R, Park M, Rousseau S, Ferri LE (2020) Activation of the pattern recognition receptor NOD1 augments colon cancer metastasis. Protein Cell 11:187–201 31956962 10.1007/s13238-019-00687-5 PMC7026222

[44] Wei X, Ye J, Pei Y, Wang C, Yang H, Tian J, Si G, Ma Y, Wang K, Liu G (2022) Extracellular vesicles from colorectal cancer cells promote metastasis via the NOD1 signalling pathway. J Extracell Vesicles 11:e12264 36068649 10.1002/jev2.12264 PMC9448875

[45] Li H, Chang X, Wang H, Peng B, Wang J, Zhang P, Zhang L (2022) Identification of a prognostic index system and tumor immune infiltration characterization for lung adenocarcinoma based on mRNA molecular of pyroptosis. Front Med 9:934835 10.3389/fmed.2022.934835 PMC9520088 36186792

[46] Möckelmann N, von Schönfels W, Buch S, von Kampen O, Sipos B, Egberts JH, Rosenstiel P, Franke A, Brosch M, Hinz S, Röder C, Kalthoff H, Fölsch UR, Krawczak M, Schreiber S, Bröring CD, Tepel J, Schafmayer C, Hampe J (2009) Investigation of innate immunity genes CARD4, CARD8 and CARD15 as germline susceptibility factors for colorectal cancer. BMC Gastroenterol 9:79 19843337 10.1186/1471-230X-9-79 PMC2776017

[47] Bertin J, Nir WJ, Fischer CM, Tayber OV, Errada PR, Grant JR, Keilty JJ, Gosselin ML, Robison KE, Wong GH, Glucksmann MA, DiStefano PS (1999) Human CARD4 protein is a novel CED-4/Apaf-1 cell death family member that activates NF-kappaB. J Biol Chem 274:12955–12958 10224040 10.1074/jbc.274.19.12955

[48] Caruso R, Warner N, Inohara N, Núñez G (2014) NOD1 and NOD2: signaling, host defense, and inflammatory disease. Immunity 41:898–908 25526305 10.1016/j.immuni.2014.12.010 PMC4272446

[49] Li YY, Pearson JA, Chao C, Peng J, Zhang X, Zhou Z, Liu Y, Wong FS, Wen L (2017) Nucleotide-binding oligomerization domain-containing protein 2 (Nod2) modulates T1DM susceptibility by gut microbiota. J Autoimmun 82:85–95 28592385 10.1016/j.jaut.2017.05.007 PMC8284907

[50] Sun X, Jobin C (2014) Nucleotide-binding oligomerization domain-containing protein 2 controls host response to *Campylobacter jejuni* in Il10-/- mice. J Infect Dis 210:1145–1154 24620022 10.1093/infdis/jiu148 PMC4168300

[51] Rommereim LM, Akhade AS, Dutta B, Hutcheon C, Lounsbury NW, Rostomily CC, Savan R, Fraser IDC, Germain RN, Subramanian N (2020) A small sustained increase in NOD1 abundance promotes ligand-independent inflammatory and oncogene transcriptional responses. Sci Signal 13:eaba3244 33293463 10.1126/scisignal.aba3244 PMC7853416

[52] Usluoglu N, Pavlovic J, Moelling K, Radziwill G (2007) RIP2 mediates LPS-induced p38 and IkappaBalpha signaling including IL-12 p40 expression in human monocyte-derived dendritic cells. Eur J Immunol 37:2317–2325 17578844 10.1002/eji.200636388

[53] Lu C, Wang A, Dorsch M, Tian J, Nagashima K, Coyle AJ, Jaffee B, Ocain TD, Xu Y (2005) Participation of Rip2 in lipopolysaccharide signaling is independent of its kinase activity. J Biol Chem 280:16278–16283 15691841 10.1074/jbc.M410114200

[54] Gong Q, Long Z, Zhong FL, Teo DET, Jin Y, Yin Z, Boo ZZ, Zhang Y, Zhang J, Yang R, Bhushan S, Reversade B, Li Z, Wu B (2018) Structural basis of RIP2 activation and signaling. Nat Commun 9:4993 30478312 10.1038/s41467-018-07447-9 PMC6255760

[55] Girardin SE, Tournebize R, Mavris M, Page AL, Li X, Stark GR, Bertin J, DiStefano PS, Yaniv M, Sansonetti PJ, Philpott DJ (2001) CARD4/Nod1 mediates NF-kappaB and JNK activation by invasive *Shigella flexneri*. EMBO Rep 2:736–742 11463746 10.1093/embo-reports/kve155 PMC1083992

[56] Inohara N, Koseki T, del Peso L, Hu Y, Yee C, Chen S, Carrio R, Merino J, Liu D, Ni J, Núñez G (1999) Nod1, an Apaf-1-like activator of caspase-9 and nuclear factor-kappaB. J Biol Chem 274:14560–14567 10329646 10.1074/jbc.274.21.14560

[57] Gutierrez O, Pipaon C, Inohara N, Fontalba A, Ogura Y, Prosper F, Nunez G, Fernandez-Luna JL (2002) Induction of Nod2 in myelomonocytic and intestinal epithelial cells via nuclear factor-kappa B activation. J Biol Chem 277:41701–41705 12194982 10.1074/jbc.M206473200

